# Evaluation of Soybean Ingredients in Pet Foods Applications: Systematic Review

**DOI:** 10.3390/ani14010016

**Published:** 2023-12-19

**Authors:** Hee S. Kim, Evan C. Titgemeyer, Erica Curles, Livia M. Olsen, Charles G. Aldrich

**Affiliations:** 1 Department of Grain Science and Industry, Kansas State University, Manhattan, KS 66506, USA; heeseong@ksu.edu; 2 Department of Animal Sciences and Industry, Kansas State University, Manhattan, KS 66506, USA; etitgeme@ksu.edu; 3 Smithbucklin Corporation, Chesterfield, MO 63017, USA; ecurles@smithbucklin.com; 4 K-State Libraries, Kansas State University, Manhattan, KS 66506, USA; livia@ksu.edu

**Keywords:** animal nutrition, antinutritional factors, digestibility, dogs, fermentability, hydrolyzed soybean, soybean protein, soybean hull, SWOT analysis

## Abstract

**Simple Summary:**

Soybean is a dominant oilseed in the U.S. Although soybeans are valuable ingredients for dogs and cats, soybean use in current pet foods has been low. The research was conducted to answer this question: What effects, if any, do soybean ingredients in dog or cat diets have on animal health and nutrition, palatability, feeding behavior, allergenicity, and extrusion processing? We summarized the most current research on soybeans in pet foods published since 2000. We discussed the strengths, weaknesses, opportunities, and threats of soybean in pet food applications. We concluded that various food processing technologies and the versatility of soybean ingredients have been demonstrated to offer considerable potential for inclusion as oil, protein, fiber, or functional ingredients in pet foods. Our work will be valuable, providing research status and gaps.

**Abstract:**

Soybean use has been low in pet foods, even though they are an excellent source of protein, polyunsaturated fatty acids, and gut fermentable fibers. The purpose of this evaluation was to conduct a systematic review of the public literature to explore how soybeans have been researched for pet food applications since 2000 and to provide strengths, weaknesses, opportunities, and threats for soybeans in the pet food industry. The review covered a total of 44 articles related to soybean ingredients and their potential value in the pet food arena. The articles were categorized by their research contents and narratively summarized to demonstrate useful information to both the pet and soybean industries. When soybean-based products have been adequately processed to reduce the antinutritive factors, they are comparable to processed animal proteins in nutritional value, palatability, and functionality in pet food processing. We conclude that various food processing technologies and the versatility of soybean ingredients allow soybean to have considerable inclusion potential in pet foods. More research on dietary soybean ingredients regarding pet food processing, fermentation benefits on health, and consumer acceptance will be needed to understand soybean’s position in the future pet food industry.

## 1. Introduction

Soybean ingredients including soybean flour, soybean meal, soybean protein concentrates, soybean oil, and other variations used in U.S. pet food totaled 534,069 tons in 2019, representing approximately 6.18% of the total 8,646,211 tons of pet food ingredients [1]. Based on quantities of total plant-related pet food ingredients, soybean meal was the third largest ingredient (427,155 tons) following corn (1,283,674 tons) and corn gluten meal (476,649 tons) [1]. Because cats are carnivores whereas dogs are omnivores, the soybean meal and soybean oil volumes used in dog foods (344,751 and 2414 tons) were greater than that in cat foods (82,404 and 479 tons), according to IFEEDER (2020). However, the soybean flour volume used in dog foods (30,912 tons) was similar to that in cat foods (32,528 tons) [1].

Soybeans are an excellent source of protein, polyunsaturated fatty acids (n-6 and n-3 fatty acids), and dietary fiber. Whole soybean contains approximately 38% crude protein (CP), 21% acid hydrolyzed ether extract (AHEE), and 20% total dietary fiber (TDF) on a dry matter (DM) basis [2]. To increase the nutritional value of soybean for different uses, various production processes are employed. According to the National Research Council (2006), soybean hulls contain ~13% CP, soybean meal contains ~44% CP, and soybean meal without hulls contains ~48% CP. Additionally, soybean protein concentrate (SPC) contains about 70% CP, and soybean protein isolate contains about 90% CP [3]. Either dehulled or whole soybeans go through an oil extraction process (solvent or mechanical), and the residues are processed in different ways to yield desirable soybean ingredients.

Soybeans contain antinutritional factors including trypsin inhibitors, urease [4], and oligosaccharides (raffinose and stachyose) [5], which may limit their use in pet food. However, most dry pet foods are produced using an extrusion process, and the heat processing deactivates most protease inhibitors [6]. Even though research and industry has demonstrated that soybeans have real value in pet diets, pet owners remain skeptical of soybean’s inclusion in pet foods due to its undeserved reputation as a poor-quality ingredient, according to many social media outlets. In other words, there is a gap in the translation of information to consumers regarding the quality of soybean ingredients in companion animals’ diets.

Thus, this research asks the question: what effects, if any, do soybean ingredients in dog or cat diets have on animal health and nutrition, palatability, feeding behavior, allergenicity, and extrusion processing? The most comprehensive review regarding soybean use in pet foods dates back to Drackley (2000) [7]. Therefore, the objective of this systematic review was to explore the original research published since 2000 regarding the use of soybean in pet foods and to summarize the findings regarding its nutritional value. This review intends to determine whether there are issues that need to be addressed regarding soybean ingredients and their value to pet diets, or if the data are available and need to be better communicated to the public.

## 2. Materials and Methods

### 2.1. Study Protocol

This systematic review of the public literature was undertaken to evaluate original research publications related to soybean consumption by companion animals (dogs and cats) in the fields of nutrition, immunology and allergenicity, behavior, and pet food processing. The study criteria were developed by the authors, and research began in October 2022. Procedurally, this work was conducted similarly to the recent article by Vanelli et al. (2021) [8].

### 2.2. Source and Research Information

The published research studies included in this review were found through searches in scientific databases. The searches were performed using the Web of Science Core Collection, CAB Direct, and Scopus via a series of developed keywords utilizing Boolean search terms. In the three databases selected, searches were conducted for documents that contained the terms (soy*) AND (dog* OR canine OR feline OR pet*) within the article title and (process* OR antinutritional OR oligosaccharide* OR extru* OR nutri* OR sustainability OR digestibility OR allergen* OR trypsin* OR ferment* OR immuno* OR gmo OR vegetarian) AND (food* OR feed*) within all fields. The last data search was performed on 4 October 2022.

### 2.3. Selection of Studies and Construction of Databases

Only journal articles written in English and published since 2000 were selected. The following materials were excluded: book references, book chapters, and literature reviews. After duplicate removal, a total of 74 articles were selected for screening by reading titles and abstracts. Only the articles featuring dogs or cats that had a soybean component as a dietary treatment were selected.

After the qualified studies were selected, the articles were tracked by a citation manager software, Zotero (6.0.27). The articles were thoroughly read and reviewed to categorize them by their research topic. The categories were animal health and nutrition, palatability, feeding behavior, allergenicity, and processing applications.

## 3. Results

### 3.1. Search and Selection of Studies

**Figure 1:**
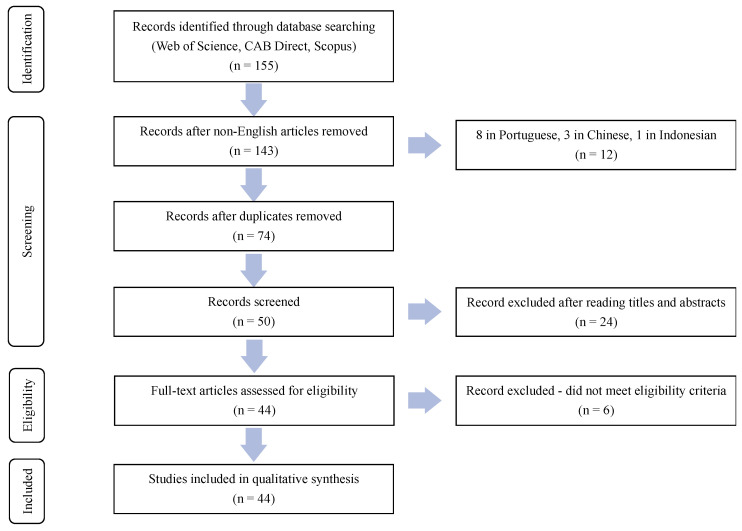
PRISMA flow diagram that identifies the total number of articles initially surveyed, and the number of articles included and excluded for this systematic review.

The results were organized into groups according to which search and scientific database they resulted from, totaling 155 articles (Figure 1). The largest number of articles (66) were identified in the Scopus database, followed by CAB Direct (51), and Web of Science Core Collection (38). The review of the articles began with the exclusion of non-English articles, which resulted in 143 papers to be evaluated. Twelve articles were excluded for language: eight written in Portuguese, three written in Chinese, and one article written in Indonesian. Next, 69 duplicate articles were removed. Of the 74 remaining articles, 24 were excluded after the title and abstracts were read. Twenty-three of the discarded articles were not related to dogs or cats, but rather the search included them due to partial word matches (for example, ‘en“dog”enous’). Additionally, one article was not related to soybeans. Finally, the remaining 50 papers were read in full. Only 44 were consistent with the established selection criteria and were evaluated in this review. Six addressed ex-vivo blood work that did not have soybean as a dietary component.

The remaining 44 articles were categorized by their research focus: animal health and nutrition (*n* = 30), palatability (*n* = 7), feeding behavior (*n* = 2), immunology and allergenicity (*n* = 10), and processing applications (*n* = 4). Some articles (*n* = 7) evaluated both the nutrition and palatability of soybean ingredients by pets, while others (*n* = 2) evaluated both the nutrition and processing parameters of soybean-including diets. There were two cat digestibility experiments studying mixed-breed cats [9] and shorthair cats [10], and [9] contained studies on both dogs and cats. There were 40 dog studies with various breeds of dogs: purpose-bred dogs [11], mongrel dogs [12,13,14], mix-breed dogs [15], Spitz dogs [16,17], beagles [4,9,18,19,20,21,22,23], Labradors [24], hound dogs [25,26], and a mix of breeds including Labrador, American Pit Bull Terrier, Weimaraner, Border Collie, Dachshund, and Great Dane [27]. The remaining three studies examined only processing of pet food or the analysis of soybean phytoestrogen concentration (soybean isoflavones) in dog foods, with no experimental animals used.

### 3.2. Soybean Ingredients Used in Pet Food

Various soybean ingredients are used in pet foods depending on the processing and nutritional composition requirements (Table 1). Different techniques for processing soybean ingredients are well described in a chapter by Rhee (2000) in “*Soy in Animal Nutrition*” and Félix et al. (2013a) [19,28]. Soybean meal is produced by submitting dehulled ground soybeans to conditioning, flaking, oil extraction, desolventization (residual hexane removal), and heating. Soybean protein concentrate is obtained from soybean meal by treating it with an ethanol solution to remove soluble sugars and increase the concentration of the protein. In contrast, soybean protein isolate is made by separation of the protein from both soluble and insoluble carbohydrates using alkaline pH adjustment, centrifugation, precipitation, drying, and membrane filtration [29]. Hydrolyzed soybean protein is obtained by enzymatically hydrolyzing defatted soybean flakes using protease to increase protein solubility and hypoallergenicity. Micronizing is a process that cooks soybeans with the heat generated by vibrating molecules under infrared light.

Soybean products (reported as soybean flour, raw soybeans, micronized whole soybeans, and toasted soybeans) analyzed and reported in manuscripts included in this review contained on average 920 ± 24.9 g/kg dry matter (DM), 51 ± 9.5 g/kg ash, 421 ± 66.6 g/kg CP, 194 ± 82.9 g/kg AHEE, and 42 ± 22.2 g/kg crude fiber on a DM basis. Soybean meal products (reported as soybean meal, low-oligosaccharide low-phytate soybean meal, regular soybean meal, high-protein soybean meal, and defatted soybean meal) used in the reviewed pet food studies had on average 908 ± 38.0 g/kg DM, 60 ± 12.5 g/kg ash, 462 ± 141.9 g/kg CP, 51 ± 35.3 g/kg AHEE, and 42 ± 19.6 g/kg crude fiber on a DM basis. Soybean protein concentrate products (reported as soybean protein concentrate, hydrolyzed soybean protein concentrate, and soybean protein isolate) used in pet foods had on average 933 ± 17.7 g/kg DM, 62 ± 13.0 g/kg ash, 705 ± 79.2 g/kg CP, 17 ± 11.7 g/kg AHEE, and 37 ± 19.0 g/kg crude fiber on a DM basis.

There were seven manuscripts that reported the antinutritional factors and protein dispersibility index of their experimental soybean ingredients (Table 2). Among the antinutritional factors, urease activity was the most frequently studied in soybean ingredients (*n* = 14), followed by trypsin inhibitors (*n* = 7), with only two data values for phytate concentrations. Levels of soybean oligosaccharides such as stachyose and raffinose were reported in one manuscript [20] with five data values for various soybean ingredients. The protein dispersibility index for different soybean ingredients was reported by [18,19,20]. Each analytical value for each component varied by ingredient and by manuscript.

Soybean hulls have also been used as a soybean-derived ingredient to provide fiber in pet food (Table 3). Soybean hulls (reported as hulls sourced from different companies) reported by the manuscripts included in this review had on average 917 ± 16.3 g/kg DM, 53 ± 3.0 g/kg ash, 130 ± 22.3 g/kg CP, 743 ± 58.5 g/kg TDF, 666 ± 44.9 g/kg insoluble fiber, and 77 ± 28.9 g/kg soluble fiber on a DM basis.

### 3.3. Impact of Soybean Ingredients on Animal Health and Nutrition

#### 3.3.1. Soybean Ingredients and Nutrient Digestibility

The articles reporting research regarding animal health and nutrition were the most numerous (*n* = 31). There were 20 manuscripts that calculated the coefficient of apparent total tract digestibility (ATTD, %) of dogs fed soybean proteins (*n* = 17) or soybean oil (*n* = 1) or fecal DM of dogs to evaluate the stool quality (*n* = 18) (Table 4). Another manuscript by [34] conducted an in vitro digestibility trial for diets including soybean nuggets. Among these 21 studies, 10 compared different types of soybean ingredients (different antinutritional factor levels or differently processed ingredients) to each other or poultry meal, 9 studies evaluated the impact of different levels of inclusion of a certain soybean-derived ingredient, and 2 studies evaluated the effects of adding exogenous enzymes into diets for dogs containing soybean ingredients. There were five manuscripts [12,13,14,35,36] that reported ileal (pre-cecal) digestibility of experimental diets containing soybean in dogs (Table 5). Three of them [12,13,14] reported both ileal digestibility and apparent total tract digestibility and degradability (ATTD) of nutrients in diets. There were four manuscripts [10,31,32,37] that calculated ATTD of diets in dogs or cats when soybean hulls were included in the experimental diets (Table 6). The remaining 5 studies of the 31 animal health and nutrition manuscripts did not calculate either digestibility or stool quality but studied the effect of soybean on dogs’ health by evaluating blood profiles, body condition, or skin condition of the fed animals, in vitro fermentation, or phytoestrogen content in the dogs’ diets.

The nutrient digestibility comparison between soybean proteins and poultry meal was inconsistent among the articles. One article found no difference in DM ATTD between soy protein fractions (soybean meal, soy flour, and soy protein concentrates) and poultry meal [11]. Venturini et al. (2018) also reported no significant differences in the ATTD of DM among soybean protein concentrate, maize gluten meal, and poultry by-product meal [23]. Carciofi et al. (2009) reported that DM ATTD was greater (*p* < 0.05) for micronized whole-soybean-containing diets than for the poultry by-product meal treatment [9]. In contrast, one article observed a higher DM ATTD for poultry-meal-containing diets than for diets containing soybean protein [13]. When comparing CP ATTD of soybean-protein-containing diets to a poultry meal diet, the CP ATTD of soybean protein diets was found to be greater than that of the poultry meal diet [11], but no differences were found between CP ATTD of micronized whole soybeans and poultry by-product meal by Carciofi et al. (2009) [9], and the CP ATTD for the poultry meal diet was higher than the soybean-protein-containing foods in Yamka et al. (2005a) [13]. In addition, there were no differences in amino acid ATTD between soybean protein fractions and poultry meal [13].

Researchers compared nutrient digestibility in dogs of various types of soybean protein ingredients, and those results varied as well. For example, there was no difference in DM ATTD of soybean meal and soybean nuggets in homemade dog foods [16] or among soybean meal, red lentils, and green gram beans [24]. The ATTD of DM was greatest for soybean protein isolate, followed by soybean meal and hydrolyzed soybean protein concentrate, and least for soybean protein concentrate [20]. The ATTD of DM was greater for low-phytate soybean meal than for soybean meal in Yamka et al. (2005b) [14]. The DM ATTD for soybean meal was greater than for whole soybean (WSB) treatments (low oligosaccharide WSB, low-oligosaccharide, and low-phytate WSB) in Yamka et al. (2005a) [13]. However, micronized whole soybeans had a higher DM ATTD than soybean meal in Carciofi et al. (2009) [9]. Félix et al. (2013a) also studied micronized whole soybeans and corroborated that they yielded a higher DM ATTD than soybean meal along with defatted soybean meal, toasted soybeans, and raw soybeans in both adult dogs and growing puppies [19].

Similar to DM ATTD, CP ATTD comparisons among soybean protein ingredients varied by study. One experiment found the ATTD of CP was highest for soybean protein isolate, followed by soybean meal and hydrolyzed soybean protein concentrate, and lowest for soybean protein concentrate [20]. The CP ATTD of soybean meal was found to be similar (*p* > 0.05) to that of red lentil and green gram in dogs fed twice daily [17,24]. Yamka et al. (2005a) reported higher CP ATTD of soybean meal than low-oligosaccharide whole soybeans and low-oligosaccharide and low-phytate whole soybeans [13], whereas Carciofi et al. (2009) reported no differences in CP ATTD between micronized WSB and soybean meal [9]. The digestibility of CP was lower for soybean meal than soybean nuggets, potentially due to the higher crude fiber content in the soybean meal diet [16]. Amino acid digestibility was similar among treatments (low-oligosaccharide, low-phytate soybean meal, conventional soybean meal, low-oligosaccharide, low-phytate WSB, and conventional WSB) except for tryptophan and histidine [14]. Tryptophan and histidine digestibilities were greater in WSB compared to low-oligosaccharide, low-phytate WSB [14]. The ATTD of several essential amino acids (isoleucine, phenylalanine, and tryptophan) in soybean meal was higher than the two WSB treatments (low-oligosaccharide WSB and low-oligosaccharide low-phytate WSB), whereas there was no difference in ATTD of nonessential amino acids [13].

As we compared the nutrient digestibility data when different inclusion levels of soybean protein ingredients were fed to dogs, the DM and CP ATTD increased in some studies [18,26] with the increase of soybean ingredients in formulas; however, those digestibilities decreased in a greater number of studies [4,12,22,34,36] as the soybean inclusion level increased. The inclusion level above which the decrease in nutrient digestibility began varied by studies (14%, [36]; linear decrease, [12]; 48%, [22]; linear decrease, [4]; 5%, [34]).

Marx et al. (2015) studied the effects of soybean oil as a fat source at different levels in dry extruded dog food [27]. They reported the ATTD of ether extract was greater for the soybean oil than beef tallow-coated diets when the fat source inclusion level was 13% [27].

From both studies that evaluated the effects of supplementing soybean meals with exogenous enzymes on nutrient digestibility, supplemental β-mannanase (5 g/kg) had no effect (*p* > 0.05) on ATTD of DM and nitrogen [15], and various combinations of protease, cellulase, pectinase, phytase, beta-glucanase, and xylanase also had no effect (*p* > 0.05) on ATTD of several nutrients [21].

Four manuscripts that reported the effect of the inclusion of soybean hulls in diets for dogs on ATTD are presented in Table 6. All four studies reported lower DM digestibility (either ileal digestibility or ATTD) in the dogs and cats fed fiber-containing diets versus no-fiber diets. Sabchuk et al. (2017) reported that the ATTD of DM, CP, and AHEE linearly decreased in dogs, as the inclusion of soybean hulls increased (from 0 to 16%) [32]. The dogs and cats exhibited similar (*p* > 0.05) DM digestibilities when fed beet pulp- and soybean-hull-containing diets [10,31,37]. Burkhalter et al. (2001) evaluated the effects of soybean hulls containing different ratios of insoluble:soluble fiber (I:S) on nutrient digestibility using ileally cannulated dogs [31]. Ileal digestibility of DM and organic matter had quadratic effects as I:S increased, having the highest digestibility when I:S ratios were highest (7.21 and 5.18) and when I:S was lowest (1.86), compared to when I:S was intermediate (2.65 and 3.17). Total tract digestibility of DM was not affected (*p* > 0.05) by the I:S ratio among the soybean hull treatments.

#### 3.3.2. Soybean Ingredients and Selected Blood Indices

Five studies evaluated blood chemistry along with nutrient digestibility in dogs fed soybean ingredients [9,16,17,21,26]. There was no significant influence of feeding diets including either soybean meal or soybean nuggets on the blood metabolic profile of the dogs in [16], with most of the parameters falling within normal ranges. Menniti et al. (2014) also reported that all serum biochemical and hematological components were within normal physiological ranges for healthy, adult dogs when they were fed experimental diets containing from 0 to 17% soybean meal [26]. Blood levels of hemoglobin and hematocrit in dogs did not change from the pre-experimental values when soybean meal was included in diets [17]. According to Carciofi et al. (2009), postprandial blood incremental urea and urea peak concentrations of dogs fed micronized whole soybeans, soybean meal, and poultry by-product meal did not differ; however, time to urea peak was delayed in dogs fed the micronized whole soybeans diet [9]. In contrast, the postprandial incremental urea and the maximum value of urea increment were higher for dogs fed soybean-meal-based diets than for dogs fed poultry-meal-based diets in the first experiment reported by Tortola et al. (2013) [21]. They found a quadratic reduction in the postprandial incremental urea with exogenous enzyme addition to the soybean meal diets in their second experiment [21]. In Menniti et al. (2014), which evaluated the effect of dietary inclusion levels of soybean meal on dogs, quadratic responses for urea nitrogen and urea nitrogen:creatinine were found with increasing soybean meal inclusion, with peaks occurring when the diet contained 6% soybean meal. Still, those parameters remained within the reference range for normal adult dogs [26].

Scheraiber et al. (2016) evaluated the effects of dietary soybean hulls (0 or 16% inclusion) on the blood biochemical profiles and the body condition of dogs. The addition of soybean hulls (replacing corn) in the diet did not change blood profiles; however, it decreased the deposition of lipids in subcutaneous tissue in dogs [38]. Oh et al. (2019) evaluated the general health, blood lipid levels, and skin condition in dogs given a dietary soybean lecithin supplement [39]. They reported no changes (*p* > 0.05) in blood profiles but did find improvement in the amount of exercise and skin exfoliation, suggesting soybean lecithin could be a nutraceutical based on the positive effect on the dogs’ general health condition. They noted the necessity of further studies to establish the appropriate dose level and administration frequency of soybean lecithin in dogs. Proot et al. (2009) fed low-protein diets with either soybean protein isolate or dehydrated poultry meat protein as their main protein source to dogs diagnosed with congenital portosystemic shunts and evaluated their blood profiles to check liver function [40]. Both low-protein diets showed improvements in the hepatic encephalopathy score, but the soybean protein isolate diet group had lower plasma ammonia than the poultry-meat protein-containing diet, suggesting better support of liver function by soybean protein isolate in dogs.

#### 3.3.3. Soybean Ingredients on Fecal Fermentative Characteristics

There were fewer studies (*n* = 9) that measured fecal pH or fermentative end products (Table 7) than studies that calculated nutrient digestibility (*n* = 21) or fecal DM (*n* = 19) when dogs were fed soybean protein ingredients (Table 4). Among the nine studies, six manuscripts reported the fecal ammonia concentration [16,19,20,21,22,23], three manuscripts presented the fecal short-chain fatty acid concentration [16,21,22], and one manuscript documented the fecal branched-chain fatty acid, phenol, and indole concentrations [22]. There were four studies that measured fecal pH or fermentative end products when dogs were fed soybean hulls (Table 8) [10,32,37,41].

Tortola et al. (2013) found that the inclusion of soybean meal in comparison to poultry meal decreased fecal DM and increased fecal output [21]. Fecal acetate, propionate, and total short-chain fatty acids (SCFA) concentrations were higher when the dogs were fed soybean-meal-containing diets than with the poultry meal diet, which indicated an increase in hindgut fermentation activity with the soybean meal treatment. When they added different kinds of exogenous enzymes to the soybean-meal-containing diets in their second experiment, fecal acetate, propionate, total SCFA, and lactate concentrations increased. They also observed higher fecal pH and fecal ammonia concentrations in dogs consuming a poultry meal diet compared to those fed soybeans. Fecal ammonia is one factor responsible for foul fecal odor [42] and a lower fecal pH is also associated with increased hindgut fermentation, which supports normal functioning of the large bowel [43]. In addition, soybean meal consumption by dogs had no effect on fecal bacteria composition [21].

Pawar and Pattanaik (2009) reported more fecal lactate, acetate, propionate, and total SCFA in dogs fed a soybean meal diet compared to a soybean nugget diet, likely due to the higher crude fiber content in the soybean meal diet [16]. There were no differences in the other measured fecal characteristics, including fecal score, fecal DM, fecal pH, and fecal ammonia concentration between the two treatments. The feces of the dogs fed the soybean-protein-isolate-containing diet had higher pH and DM content, and those dogs produced less feces on a fresh matter basis than other dogs fed soybean meal or soybean protein concentrates [20]. Fecal ammonia content was not influenced by the dietary soybean protein ingredients [20]. In addition, Félix et al. (2013b) reported that dietary soybean meal resulted in the highest intestinal gas production, but there were no differences among the other dietary soybean protein ingredients [20].

Beloshapka et al. (2016) reported the total dietary fiber content increased (5.70 to 13.13% on DM basis) in dog diets as the bioprocessed soybean protein inclusion level increased from 0 to 48% [22]. Fresh fecal DM was lower and fecal acetate, propionate, and total SCFA concentrations were greater for dogs fed the 24 and 48% soybean protein treatments compared with dogs fed the 0% soybean protein diet. Fecal output was greater for dogs fed the 48% soybean protein treatment; however, fecal pH was not affected by dietary soybean protein inclusion. Fecal ammonia, isovalerate, isobutyrate, total branched-chain and minor fatty acid concentrations, phenol, and indole concentrations were lower for dogs fed 48% soybean protein than the control. Phenols and indoles, like ammonia, can worsen fecal odor, and some evidence suggests they have a negative impact on intestinal health [44].

When comparing the effect of soybean hulls to other fiber sources, the fecal DM content of the dogs fed soybean hulls was lower than of the dogs fed sugarcane or cellulose, but higher than for those fed beet pulp [32]. However, intestinal gas score and intestinal gas production area, measured by radiographic images taken before and after the test diet was fed, were not influenced (*p* > 0.05) by dietary fiber sources [32]. Myint et al. (2017) compared the effects of supplementing soybean hulls or cellulose to dogs [41]. Dietary soybean hulls in dogs decreased fecal pH compared with cellulose, with higher fecal total SCFA, acetate, butyrate, and lactate concentrations [41]. Detweiler et al. (2019a) also reported higher total fecal SCFA concentrations in dogs fed soybean hulls or beet pulp than in dogs fed cellulose or a no-fiber diet [37]. The fecal indole and skatole concentrations in dogs fed the soybean hull diet were lower than the cellulose diet, whereas fecal ammonia concentration was unaffected [41]. They also found that soybean hull supplementation led to a higher relative proportion of total lactobacilli, which can lower intestinal pH in dogs’ feces than cellulose supplementation [41]. Detweiler et al. (2019b) evaluated the effect of dietary fiber sources on cats and found no differences in fecal ammonia or total phenol and indole concentrations among treatments (no fiber, beet pulp, cellulose, and soybean hulls) [10]. In addition, cats fed beet pulp had greater total fecal SCFA concentrations, followed by soybean hull and no fiber, with the lowest for cellulose treatment.

Yamka et al. (2006) evaluated the flatulence and fecal odor metabolites of dogs fed low-oligosaccharide low-phytate soybean meal, conventional soybean meal, or poultry by-product meal diets with or without supplemental β-mannanase [15]. They reported no difference in flatulence or fecal odor metabolites such as indoles, phenols, and volatile sulfur-containing compounds when measured by a solid-phase microextraction procedure with gas chromatography, regardless of supplemental enzyme; however, the different dietary protein sources did affect the fecal odor metabolites but not the flatulence. Although dogs fed poultry by-product meal had low fecal output, the fecal odor metabolites excreted per day were greater than for dogs consuming low-oligosaccharide low-phytate soybean meal or conventional soybean meal diets. These data suggest that dogs fed poultry by-product meal as a dietary protein source had feces that contained more unpleasant odor components than soybean protein-fed dogs [15].

### 3.4. Soybean Ingredients on Palatability

There were six studies that evaluated the palatability of soybean ingredients using dogs [16,17,18,22,23,32], whereas there was one study that assessed the palatability of soybean ingredients in cats [9]. Two studies assessed the palatability of the foods to the dogs using a 1–4-point scale (1 = ate an entire meal without hesitation, 4 = refused to eat), and the other five studies used the two-bowl method, which measures preference of one food over another by presenting two foods to dogs and recording the total quantity of each food consumed. Pawar and Pattanaik (2009) did not find a significant difference (*p* > 0.05) between the dietary treatments (soybean meal or soybean nugget inclusion) in palatability to dogs [16]. Pattanaik and Kore (2021) reported the palatability score of the experimental diets (soybean meal, red gram, or lentil inclusion) using the same 1–4-point scale [17]. The palatability score was similar (*p* > 0.05) among the treatments.

Félix et al. (2012) measured the palatability of dog diets using a pair-wise diet comparison for two consecutive days [18]. They made six comparisons to evaluate the effects of enzymes and the type and level of soybean meal on diet palatability. Dogs consumed more regular soybean-meal-containing diets (either 15 or 30%) than the control, which contained higher poultry offal meal and maize with no soybean meal. The study also reported the 30% regular soybean meal diet with enzymes included was preferred over the control or 30% regular soybean meal diet without enzymes. Beloshapka et al. (2016) performed two-bowl tests once daily for two days in a row to evaluate the palatability of bioprocessed soybean protein to dogs [22]. Based on the intake ratios from the experiments, they reported that the optimal inclusion of the bioprocessed soybean protein was 12% with greater consumption by the dogs compared to the 0% control. Sabchuk et al. (2017) evaluated the palatability of dog foods containing sugarcane, beet pulp, cellulose, and graded levels of soybean hulls using the pair-wise diet comparison method for two consecutive days [32]. There were no differences in food preference among the tested diets (reference to 4% soybean hull, reference to 16% soybean hull, reference to 13.1% sugar cane, reference to 16% beet pulp, and reference to 12.1% cellulose). Venturini et al. (2018) evaluated the palatability of dog diets containing poultry by-product meal, maize gluten meal, or soy protein concentrate using the two-bowl test method [23]. Dogs preferred the poultry by-product meal over the maize gluten meal diet. There were no differences in preference between poultry by-product meal and soybean protein concentrate diets or soybean protein concentrate and maize gluten meal diets.

Carciofi et al. (2009) evaluated the palatability of experimental foods to cats using the two-bowl test method on three consecutive days, comparing the relative consumption of two diets (micronized whole soybeans or corn gluten meal inclusion) [9]. They found that the cats preferred the diet containing micronized whole soybeans over the maize gluten meal diet with a twofold greater consumption rate.

### 3.5. Soybean Ingredients on Dog Behavior

Sabchuk et al. (2014) evaluated dogs’ behavior for 24 h, recording the frequency of occurrence for each behavior while feeding diets with or without soybean hulls [33]. There were no differences in the dogs’ behavior with dietary soybean hull inclusion. Similarly, Scheraiber et al. (2018) evaluated dog behavior after eating diets with or without soybean hulls (0 or 16%) [45]. They observed a reduction in scratching behavior and stereotypical behavior (repetitive regular movements) (*p* < 0.10) in animals fed a diet containing soybean hulls [45].

### 3.6. Soybean Ingredients on Allergenicity and Immunology

There were six studies [46,47,48,49,50,51] evaluating the effects of hydrolyzed soybean protein on immunologic responses by challenged dogs. The work by [46] demonstrated significant pruritus (itchy skin) after an oral challenge with soybean protein but not with hydrolyzed soybean protein. The soybean and corn-specific serum IgE did not increase in dogs post challenge. Similarly, Serra et al. (2006) found a significant reduction in soybean-specific IgE binding to the hydrolyzed soybean protein than to the native soybean protein in serum obtained from dogs with soybean hypersensitivity [47]. Puigdemont et al. (2006) observed no response to oral administration of hydrolyzed soybean protein in dogs with soybean hypersensitivity [48]. Moreover, Biourge et al. (2004) reported that dogs diagnosed as having an adverse food reaction or a combined adverse food reaction and atopy showed a decrease in the pruritus score after 2 months of feeding the soybean-hydrolysate-containing diets [49]. Vandresen and Farias (2018) also reported on the pruritus score and the Canine Atopic Dermatitis Lesion Index, and they observed that the hydrolyzed soybean dog food was effective at partially reducing clinical signs of food-induced atopic dermatitis [50], whereas the homemade food group did not (*p* > 0.05) present improvements. In addition, Biourge and Fontaine (2004) reported that a soybean-hydrolysate-based diet could significantly improve the clinical conditions, fecal score, pruritus score, and skin lesions of dogs suffering both from exocrine pancreatic insufficiency and skin disease [51].

Willis-Mahn et al. (2014) evaluated soybean antigens in dry dog foods that have a “no-soybean” claim and veterinary therapeutic dry dog foods designed for food elimination trials using enzyme-linked immunosorbent assay (ELISA) testing [52]. They detected a positive response for soybean antigens in three of the four “no-soybean”-claiming diets and four of the seven veterinary therapeutic diets. They concluded that a veterinary therapeutic diet should be carefully chosen to treat soybean food adverse reactions in dogs. Mikawa et al. (2021) investigated the effects of oral administration of a fermented soybean product, natto, on the cellular immune activity of dogs [53]. They reported that dietary natto increased the cytotoxic activity of peripheral natural killer cells and the expression of TNF-α in peripheral blood mononuclear cells after an antigen stimulation in dogs. They concluded that dietary natto might be beneficial in augmenting cellular immune activity in dogs.

In addition, Cerundolo et al. (2004) determined the phytoestrogen content in commercial dog foods that contained soybeans or soybean fractions and foods without any soybean-related ingredients listed on the label [54]. They found that most of the diets that included soybean ingredients had detectable concentrations of phytoestrogens, which could have biological effects when ingested by dogs long-term. To explore that possibility, Cerundolo et al. (2009) evaluated the effect of dietary soybean isoflavones on general health and adrenocortical and thyroid gland function in dogs [55]. They were fed a hydrolyzed soybean-isolate-based diet or the same diet without isoflavones, and most serum concentrations of hormones were not affected by diet. However, they concluded that feeding soybean to dogs on a long-term basis may influence endocrine function due to the phytoestrogens, although more studies are needed to confirm or refute this supposition.

### 3.7. Soybean Ingredients on Pet Food Processing Application

There were four studies [23,30,34,56] that addressed pet food processing attributes and two [4,19] that evaluated the impact of extrusion processing on antinutritional factors of soybean ingredients in dog diets.

Purushotham et al. (2007) attempted to optimize steam-conditioning and extrusion operations to inactivate antinutritional factors in soybeans for pet food applications [30]. They demonstrated that urease activity and trypsin inhibitor levels decreased (2.0 and 50 mg/g to 0.1 and 5 mg/g, respectively) as the extrusion temperature increased to 120 °C. Extrusion of soybeans between 120 and 140 °C did not affect major nutrient compositions but did improve nutritional value through the inactivation of antinutritional factors. Urease activity was reduced in all diets containing 30% soybean protein products (defatted soybean meal, soybean meal, micronized soybeans, toasted soybeans, and raw soybeans) after extrusion, but trypsin inhibitor activity was reduced only in the diets containing defatted soybean meal, soybean meal, and raw soybeans [19]. Urease and trypsin inhibitor activity in the diets increased with the inclusion of raw soybeans up to 30% before and after extrusion [4]. Félix et al. (2020) also reported a decrease in antinutritional factor activity after extrusion [4]. Kaur et al. (2021) prepared dog food using soybean nuggets with three processing methods [34]: raw, boiled, and extruded, and then calculated the in vitro digestibility of nutrients. They concluded that the extrusion improved the digestibility of DM, CP, ether extract, and organic matter.

Venturini et al. (2018) evaluated the effect of soybean protein concentrate at different inclusion levels up to 45% on extrusion processing and kibble macrostructure [23]. The substitution of poultry by-product meal by coarse soybean protein concentrate increased extrusion motor load, temperature, die pressure, and specific mechanical energy. With the increase of coarse soybean protein concentrate in the dog diets, the bulk density of the kibble, specific length, and radial expansion rate after dryer decreased, whereas the starch gelatinization increased. In summary, soybean protein concentrate demonstrated good functionality during the extrusion processing and improved kibble expansion and starch gelatinization.

Lyng et al. (2022) investigated the effect of independent extrusion process variables when producing pet food extrudates containing defatted soybean flour alone or combined with beef meat or connective tissue protein (collagen fiber) [56]. They found that defatted soybean flour with water expanded less after extrusion and could not retain a chunk-like appearance after retorting. However, the defatted soybean flour combined with beef meat or connective tissue expanded more and retained its pre-retort paste-like structure after retorting. Overall, they indicated that a combination of formula and extrusion process parameters has a significant effect on the extrusion processing and the resulting product characteristics.

## 4. Discussion

### 4.1. The Strengths of Soybeans in Pet Food

Soybeans should be described beyond their nutritional chemical composition to represent their value. Based on the summarized literature, the strengths of soybeans in pet food applications are nutrition, palatability, and functional processing attributes. Macronutrients in soybeans, such as crude fat, crude protein, and crude fiber, are either digestible or fermentable for companion animals, and the nutrient profiles are comparable to poultry by-product meal. Additionally, the variety of soybean ingredients with different chemical or physical characteristics enables formulators to increase or decrease certain nutrient digestibilities, fermentability, or expansion of the processed final products.

The nutritional compositions of various soybean ingredients discussed in the literature review can be explained by the usual soybean protein processing stream. The soybean protein processing starts with dehulled full-fat soybeans, then they are defatted by oil extraction with hexane as the solvent [57]. To remove the remaining solvent from the defatted soybeans, various processing conditions in terms of heat temperature, moisture, and retention time can be used and have effects on protein denaturation related to activity of proteinaceous antinutritional factor activities [29]. The results clearly showed that soybeans contain higher fat and energy density compared to soybean protein ingredients, which are made from the defatted soybean flakes. In addition, raw soybeans contain higher urease activity and trypsin inhibitors than soybean meal or toasted soybeans. Defatted soybean flakes are used to make soybean meal, soybean protein concentrate, soybean protein isolate, hydrolyzed soybean protein, or textured soybean protein by applying different processing conditions [57]. Among soybean protein ingredients, soybean meal has a lower protein level than soybean protein concentrates because the soluble carbohydrates are extracted from defatted flakes before grinding when making soybean protein concentrate. Protein dispersibility is higher for raw soybeans and soybean protein isolate than for micronized or toasted soybeans, soybean meal, or soybean protein concentrates. Protein soluble in KOH solutions was also greater for raw soybeans and soybean protein isolate than for soybean protein concentrates or soybean meal. Soybean processing influences the protein fraction, and the extent of the soybean protein denaturation affects protein digestibility [29]. Félix et al. (2013b) reported high correlations among CP digestibility, protein dispersibility index, and soluble protein contents in KOH [20]. An unfolded protein structure can be more accessible to proteolytic digestive enzymes; however, it can also increase protein aggregation by increasing the interaction between proteins with other proteins or components, which can lower accessibility to enzymes [29]. Even though the various soybean antinutritional factors and protein denaturation also impact the protein quality of the ingredients, only a few studies have reported data on these effects in companion animals.

Numerous researchers have explored soybean for its utilization in dogs and cats by measuring nutrient digestibility, stool quality, blood chemistry, and fecal fermentative characteristics. Evaluation of the effects of dietary soybean ingredients on nutrient digestibility and stool quality has been the primary area of interest for the largest number of manuscripts published. The nutrient digestibility of soybean proteins was comparable to poultry meal, often demonstrating no significant differences between these protein sources. Although soybean protein fractions used in experiments have included soybean flour, conventional soybean meal, and soybean protein concentrates, the nutrient digestibility of soybean proteins has been mostly comparable to poultry proteins. Nutrient digestibility among various soybean protein ingredients varies based on their preparation/processing, antinutritional factor concentrations, and crude fiber contents. With increasing inclusion levels of soybean protein ingredients in canine diets, nutrient digestibility decreased more often than it increased or remained the same.

None of the studies included in this review found changes in blood chemistry beyond accepted reference values due to dietary soybean ingredients being included in diets for dogs, thus showing no deleterious effects of soybean ingredients on animal health. Some studies found fecal production increased with the inclusion of dietary soybean meal compared to poultry meal due to the higher fiber content [13,15,18], but dietary soybean meal additions also resulted in higher concentrations of fecal fermentative products such as SCFA [21,22]. In addition, putrefactive compounds in feces such as indole, skatole, and ammonia were either no different or lower in dogs fed soybean ingredients than in those fed beet pulp or no fiber. The high fermentability of soybean ingredients in dogs impacts the gut microbiota population and is beneficial for their gut health. Soybean ingredients had either no effect on or increased the palatability of pet foods in companion animals, which is promising for pet food formulators.

Soybean protein is known for excellent functional properties, such as water-holding, gelling, fat-absorbing, and emulsifying capacities in food products, which is why it is frequently used as an ingredient in extrusion [58]. The inclusion of soybean ingredients influenced the extrusion processing parameters and the expansion of dog kibbles [23]. However, there was no significant negative effect on kibble formation, which is one of the critical factors for industrial producers when choosing an ingredient for their formulas. Further studies to compare the processing functionality among various soybean ingredients or comparing soybean to other commonly used animal or plant proteins would be helpful to demonstrate the strength of soybean products in pet food processing.

### 4.2. The Weaknesses of Soybean in Pet Food

The weakness of dietary soybeans for companion animals lies in their antinutritional factors and potential allergenicity. However, there are no routine tests in normal feed use to detect antigenic or toxic activity of soybean components unless these are monitored separately [59]. Furthermore, no data are available on the variability of antigenic components between soybean varieties, source of the soybeans, or various soybean ingredients. Possible pathological effects of dietary soybean on various animals (rats, piglets, and pre-ruminant calves) were indicated by Csaky and Fekete (2004) [59] They reported that antinutritive factors such as trypsin inhibitors induced pancreas hypertrophy, lowered methionine and cysteine absorption, shortened villi in the small intestine, and reduced growth performance in animals [59]. Soybean oligosaccharides are considered antinutritional factors that may induce flatulence in dogs [60]. Intestinal gas production by dogs fed soybean meal was greater than for dogs fed a reference diet without soybean products, but there were no differences between the reference diet and diets containing either soybean protein concentrate or soybean protein isolate [20]. Another study that examined this outcome found no differences in flatulence among low-oligosaccharide low-phytate soybean meal, conventional soybean meal, and poultry by-product meal [15].

Dréau and Lallès (1999) reported that the predominant storage proteins of soybeans, glycinin and β-conglycinin, are antigens and can cause allergenic reactions in the intestinal mucosa of pre-ruminant calves and early weaned piglets [61]. All subunits from the soybean glycinin protein family were identified as soybean allergens for humans [62]. Fu et al. (2007) further identified soybean β-conglycinin α-subunit as a potential allergen for young piglets [63]. Taliercio et al. (2014) identified the β-subunit of soybean β-conglycinin as antigenic in dogs by demonstrating that peptides of the β-subunit of conglycinin were bound by IgG and IgE antibodies from canine sera [64]. However, the pathological effects on companion animal health and immunological responses to dietary soybean or soybean meal need to be studied further.

Various approaches such as food processing technology, genetic engineering, and targeted breeding have been studied to remove antinutritional factors and allergens from soybeans [63]. The activities of antinutritional factors and allergens in soybean can be adjusted by enzyme, heat, ethanol treatment, or fermentation, although most treatments leave conglycinin residues intact [59,65,66,67,68,69]. To lower adverse food reactions such as food allergies in dogs, soybean meal is often hydrolyzed and used for hypoallergenic prescription diets. The literature shows that hydrolyzed soybean protein leads to significantly fewer allergenic reactions compared to soybean or soybean meal, supporting the supposition that hydrolysis of soybean proteins overcomes these weaknesses of soybean in pet food applications and provides new opportunities [46,48,49,50]. Further research to compare allergenicity between soybean proteins and other animal proteins would be valuable. This is because the most common food allergens in dogs with diagnosed food allergies are beef, dairy, or chicken, whereas soybean is one of the least common food allergens in dogs [70].

Hot water treatment, aqueous alcohol extraction, or isoelectric protein precipitation used to manufacture soybean protein concentrates and isolates remove oligosaccharides [71], resulting in lower flatus activity. The optimal processing conditions to remove or reduce all soybean antinutritional factors, such as trypsin inhibitors, urease, lectins, conglycinin, oligosaccharides, and overall antigenicity, and to increase the protein quality should be assessed to deliver the maximal nutritional value of soybeans to pet animals. Again, selection of the correct soybean ingredient among various choices can optimize the value of soybean ingredients.

### 4.3. The Opportunities of Soybeans in Pet Food

Opportunities for soybeans are increasing with the ongoing pet food trends. As the market matures and premiumization progresses, pet food development has focused on functional health benefits and sustainability in recent years. Plant-origin proteins have been proposed to improve the sustainability of pet foods by using fewer natural resources and maintaining a smaller carbon footprint than animal-origin proteins [72,73]. The pet food industry produces more segmented products making claims such as ‘gut health’, ‘vegetarian’, ‘vegan’, ‘plant-based’, and ‘sustainable dog food’ to fit the specific needs of consumers. About 20 million vegetarian pet owners are in the United States, and 45% of pet owners (including non-vegetarian pet owners) expressed a desire to feed a plant-based diet if one were available that met their criteria [74]. There is a small but growing niche market for vegetarian pet foods. Soybean has been researched because it offers high protein and a good amino acid composition similar to that of meat [75]. Soybean protein sources had lower methionine levels than poultry meal [11], which may necessitate supplemental methionine, cystine, and taurine in the formulas to meet the amino acid requirements for dogs and cats. A recent study by Golder et al. (2020) reported no differences in digestibility between plant and animal protein in dogs and found that the plant protein had higher digestibility than animal protein in cats [76]. However, soybean products need to be adequately processed to achieve nutrient digestibility equal to meat proteins in dogs [77].

Obesity in dogs has been identified as a pressing issue that may negatively affect animal health and longevity [78]. Thirty-nine percent of dog owners and 45% of cat owners reported that their pets are overweight or obese [79]. Numerous factors such as genetics, amount of physical activity, and the energy consumption from their diet or treats are involved in pets becoming obese [80]. The feeding of high-fiber diets has been studied as a means of inducing weight loss, and it has been shown to induce weight loss and satiety in dogs [81,82]. The inclusion of soybean hulls as fiber in diets led to lesser nutrient digestibility according to this literature review, and this can dilute energy consumption from diets and increase weight loss in animals. Dietary soybean hulls decreased the deposition of subcutaneous lipids in dogs [38], providing further evidence that soybean ingredients could benefit animal health. Studies on dog behavior in response to dietary soybean hull inclusion showed either no difference or a reduction in scratching behavior with a lower metabolizable energy intake when soybean hulls were present in the diet [33,45]. Numerous studies in the literature have shown that dietary soybean ingredients increase fermentative products by the hindgut microbiome and can result in a healthy gut by acting as prebiotics in companion animals. The addition of exogenous carbohydrase enzymes to diets was not effective at increasing nutrient digestibility in dogs according to two manuscripts. However, a carbohydrase mixture did improve nutrient digestibility in pigs [83,84,85,86], which supports the potential for improved digestibility through enzyme supplementation in dogs. Traylor et al. (2001) reported that phytase supplementation improved Ca and P utilization from soybean meal in growing swine [87]. Kerr et al. (2010) also reported clear improvements in P digestibility in finishing pigs when fed phytase with soybean-meal-containing diets [88]. Further research is needed to find out the right enzymes and enzyme supplementation timing for dogs to increase the nutrient utilization of soybeans. In addition, microbially fermented soybean ingredients and phytoestrogens, such as isoflavones, were suggested to have additional potential health benefits (prevention of certain types of cancers or control of obesity), but there are also concerns about the estrogenic effect when dogs receive high amounts of isoflavones on a long-term basis [53,55]. More detailed research on the optimal dose and health effects from long-term consumption of these functional soybean-based ingredients may provide more opportunities to expand soybean utilization in pet food. In addition, there is an obvious need to conduct more studies in cats, because information regarding the effects of dietary soybean ingredients on cat nutrition or health was scarcer than that for dogs in the reviewed literature.

### 4.4. The Threats of Soybeans in Pet Food

The predominant threat to the use of soybeans is the underlying negative perceptions of soybean among some marketers and consumers. One of the top pet food marketing claims in 2021 was “no corn or soybean,” which influenced 25% of pet owners’ decisions to purchase specific pet food according to the survey conducted by the Association for Pet Obesity Prevention [79]. According to another survey (Association for Pet Obesity Prevention, 2018), pet owners were more influenced by the descriptive statements about diets when purchasing pet foods versus veterinary professionals [89]. These two surveys support the idea that any statements about soybean would cause more concern among owners than professionals, indicating the potential for misinformation to sway owners or a disconnect with the science. There are several online articles that are approachable to consumers due to their language and style that assert that soybeans are bad for dogs. Some of the reasons provided for avoiding soybean in dog foods are (1) most U.S.-grown soybeans are genetically modified organisms that contain glyphosate resistance; (2) soybean contains antinutrients such as lectins that can cause digestive issues and may lead to leaky gut syndrome; (3) soybean may lower thyroid function by goitrogenic effects; (4) soybean may trigger food allergies; (5) phytoestrogens in soybean are potentially harmful and can lead to infertility, polycystic ovarian syndrome, and breast cancer; (6) soybean is antigenic; (7) soybean contains trypsin inhibitors; and (8) soybean is high in phytic acid [90]. However, most of these reasons to avoid soybean are assumptions with no published scientific evidence. The results of this systematic review regarding dietary soybean ingredients support the positive nutritive value of soybean for companion animals. With the expansion of press reporting on and disseminating scientific research data in recent years, there are some consumer-friendly sources that try to educate consumers on the facts about soybean, including that it has high nutritional value and is well digested in dogs. One such article explains that there is no evidence that normal levels of soybean in dog foods can lead to illness [91]. Bioactive proteins such as trypsin inhibitors or lectins in soybean are denatured by the cooking process [6], and they should not be an issue in conventional processed pet foods. Pet food companies themselves, such as Purina, are attempting to address the challenge of misinformation [92]. They counter several common myths, such as explaining that soybean meal does not increase flatulence or bloat in dogs [15,93] and that soybean meal is not highly allergenic [94]. These efforts could start to shift the perception of soybean among consumers and producers, potentially making them more open-minded. The recent white paper on soybean in pet foods from ADM revealed that 80% of U.S. pet owners are open to soybean, corn, or wheat in their pets’ diet based on independent consumer surveys [95]. According to the survey, motivators for including soybean were health/nutrition, taste, and recommendations. Another white paper published by ADM supported that pet owners’ attitudes about soybean ingredients for their pets are highly open-minded, suggesting that the influence of the “no corn, soybean, or wheat” slogan that has impacted the pet food industry for nearly three decades is waning [96]. Because pet foods are commercial products, marketing slogans and perceptions of consumers and producers can significantly affect the utilization of soybean. Therefore, scientists must investigate the negative claims of soybean in pet foods and publish studies to provide valid information about soybean utilization in pet foods. Together with research publications in scientific journals and online magazines, periodic surveys that examine the general perception of soybean are also necessary to (1) determine if the scientific evidence has been communicated well to industrial stakeholders and consumers, not just with other scientists; and (2) predict the future market needs for soybean.

## 5. Conclusions

This paper comprehensively summarizes the effects of various soybean ingredients in multiple pet food applications. It provides an overview of gaps in the research where more attention is needed from future researchers in the pet food industry. Various food processing technologies have been applied to soybeans to produce ingredients that contain desired nutrients in higher concentrations. The versatility of soybean ingredients has been demonstrated to offer considerable potential for inclusion as oil, protein, fiber, or functional ingredients in pet food as strengths. Questions remain regarding the concentrations of antinutritional factors in various soybean ingredients and efficient pet food processing conditions or exogenous enzyme supplementation methods to completely overcome concerns about the weaknesses. More feeding trials on pet food processing of prepared diets with soybean ingredients are required to determine the relationship between processing and the nutritional value of the diets. More research is needed on the potentially beneficial effects of hindgut fermentation and functional fractions of dietary soybean to validate opportunities. Lastly, research that studies the effect of the inclusion of soybean ingredients in pet foods on pet owners’ perceptions, such as consumer studies, sensory analysis, or survey studies, will be needed to better understand soybean’s acceptability and overcome the threats in the future pet food industry.

## Figures and Tables

**Table 1:** Nutritional composition (g/kg on a dry matter basis, unless otherwise stated) of soybean ingredients in various pet food studies. (n.r.: not reported).

Authors	Soy Ingredient	^1^ DM	Ash	^2^ CP	^3^ AHEE	^4^ ME, kcal/g	Ca	Total P	Crude Fiber	^5^ NDF	^6^ ADF	^7^ TDF
Félix et al., 2020 [4]	Raw soybeans	907.2	45.5	398.3	207.2	413.3	n.r.	n.r.	69.7	n.r.	n.r.	n.r.
Carciofi et al., 2009 [9]	Soybean meal	861.9	34.8	479.0	21.9	n.r.	n.r.	n.r.	n.r.	n.r.	n.r.	n.r.
Carciofi et al., 2009 [9]	Micronized whole soybeans	938.3	44.6	412.5	250.5	n.r.	n.r.	n.r.	n.r.	n.r.	n.r.	n.r.
Clapper et al., 2001 [11]	Soybean meal	874.0	74.0	566.0	25.0	n.r.	n.r.	n.r.	n.r.	n.r.	n.r.	157.0
Clapper et al., 2001 [11]	^8^ Soybean flour	927.0	70.0	553.0	28.0	n.r.	n.r.	n.r.	n.r.	n.r.	n.r.	162.0
Clapper et al., 2001 [11]	^9^ Soybean protein concentrate 1	949.0	61.0	722.0	11.0	n.r.	n.r.	n.r.	n.r.	n.r.	n.r.	213.0
Clapper et al., 2001 [11]	^10^ Soybean protein concentrate 2	943.0	70.0	704.0	8.0	n.r.	n.r.	n.r.	n.r.	n.r.	n.r.	175.0
Clapper et al., 2001 [11]	^11^ Soybean protein concentrate 3	945.0	41.0	705.0	32.0	n.r.	n.r.	n.r.	n.r.	n.r.	n.r.	211.0
Yamka et al., 2006 [15]	Low-oligosaccharide low-phytate soybean meal	967.5	47.0	197.6	115.0	383.0	8.0	7.0	23.0	n.r.	n.r.	n.r.
Yamka et al., 2006 [15]	Soybean meal	970.1	48.0	204.1	117.0	382.3	8.0	7.0	27.0	n.r.	n.r.	n.r.
Félix et al., 2012 [18]	Regular soybean meal	898.8	71.2	515.1	45.1	322.0	n.r.	n.r.	73.2	n.r.	n.r.	n.r.
Félix et al., 2012 [18]	High-protein soybean meal	891.5	68.2	561.2	45.9	331.0	n.r.	n.r.	51.7	n.r.	n.r.	n.r.
Félix et al., 2013a [19]	Defatted soybean meal	939.1	61.4	524.1	26.2	336.0	4.0	9.0	16.0	89.1	n.r.	n.r.
Félix et al., 2013a [19]	Soybean meal	892.2	61.0	467.2	41.1	330.5	4.0	6.0	53.4	147.2	n.r.	n.r.
Félix et al., 2013a [19]	Micronized soybeans	957.3	47.0	408.0	215.4	435.9	2.0	6.0	15.3	139.0	n.r.	n.r.
Félix et al., 2013a [19]	Toasted soybeans	897.1	49.8	376.2	234.0	435.1	3.1	6.1	41.3	106.0	n.r.	n.r.
Félix et al., 2013a [19]	Raw soybeans	895.0	49.0	376.4	231.2	434.3	3.1	6.2	40.5	104.2	n.r.	n.r.
Félix et al., 2013b [20]	Soybean meal	902.3	62.6	556.1	36.6	326.8	5.4	6.8	56.1	170.8	85.3	n.r.
Félix et al., 2013b [20]	^12^ Soybean protein concentrate	911.1	64.5	618.8	21.5	322.4	6.6	9.0	45.2	271.4	85.0	n.r.
Félix et al., 2013b [20]	^13^ Soybean protein concentrate	917.3	60.5	687.2	18.5	321.8	5.2	9.1	46.4	282.0	87.8	n.r.
Félix et al., 2013b [20]	^14^ Hydrolyzed soybean protein concentrate	906.9	61.8	692.2	19.8	321.5	5.8	9.2	47.9	292.5	87.4	n.r.
Félix et al., 2013b [20]	Soybean protein isolate	955.9	45.1	898.0	36.9	352.5	3.3	7.1	0.6	32.2	8.5	n.r.
Venturini et al., 2018 [23]	^15^ Soybean concentrate	927.0	74.4	673.1	4.3	308.8	n.r.	n.r.	49.6	n.r.	n.r.	n.r.
Venturini et al., 2018 [23]	^16^ Soybean concentrate	940.0	81.9	643.6	4.3	312.7	n.r.	n.r.	30.9	n.r.	n.r.	n.r.
Menniti et al., 2014 [26]	Soybean meal	881.0	68.1	552.8	35.2	330.6	3.9	5.9	37.5	n.r.	n.r.	n.r.

^1^ DM = dry matter, ^2^ CP = crude protein, ^3^ AHEE = acid hydrolyzed ether extract, ^4^ ME = metabolizable energy (calculated using the predicted equations in dog foods ((8.5 × Crude fat) + (3.5 × Crude protein) + (3.5 × Nitrogen-free extract (NFE)), ^5^ NDF = neutral detergent fiber, ^6^ ADF = acid detergent fiber, ^7^ TDF = total dietary fiber, ^8^ Soybean flour = Soyafluff 200W, ^9^ Soybean protein concentrate = traditional aqueous alcohol-extracted soybean protein concentrate (Profine F), ^10^ Soybean protein concentrate 2 = extruded soybean protein concentrate (Profine E), ^11^ Soybean protein concentrate 3 = modified molecular weight soybean protein concentrate (Soyarich I), ^12^ Soybean protein concentrate = soybean protein concentrate with 600 g crude protein/kg, ^13^ Soybean protein concentrate = soybean protein concentrate with 700 g crude protein/kg, ^14^ Hydrolyzed soybean protein concentrate = soybean protein concentrate with 700 g crude protein/kg, ^15^ Soybean concentrate = coarse particle size, ^16^ Soybean concentrate = small particle size; 200 μm.

**Table 2:** Antinutritional factors (urease, trypsin inhibitor (TI)), protein dispersibility index (PDI), protein solubility in KOH (KOH), and sugar compositions (on a dry matter basis, unless otherwise stated) of soybean ingredients in various pet food studies. (n.r.: not reported).

Authors	Soybean Ingredient	Urease, ΔpH	TI, mg/g	Phytate, g/kg	Stachyose, g/kg	Raffinose, g/kg	Sucrose, g/kg	Galactose, g/kg	Fructose, g/kg	Total Sugar, g/kg	PDI, %	KOH, %
Félix et al., 2020 * [4]	Raw soybeans	1.86	15.9	n.r.	n.r.	n.r.	n.r.	n.r.	n.r.	n.r.	n.r.	89.30
Yamka et al., 2006 [15]	Low-oligosaccharide low-phytate soybean meal	n.r.	n.r.	0.7	0.1	0.1	n.r.	n.r.	n.r.	n.r.	n.r.	n.r.
Yamka et al., 2006 [15]	Soybean meal	n.r.	n.r.	1.5	22.4	2.0	n.r.	n.r.	n.r.	n.r.	n.r.	n.r.
Félix et al., 2012 * [18]	Regular soybean meal	0.05	n.r.	n.r.	n.r.	n.r.	n.r.	n.r.	n.r.	n.r.	10.70	n.r.
Félix et al., 2012 * [18]	High-protein soybean meal	0.04	n.r.	n.r.	n.r.	n.r.	n.r.	n.r.	n.r.	n.r.	10.30	n.r.
Félix et al., 2013a [19]	Defatted soybean meal	0.22	9.0	n.r.	n.r.	n.r.	n.r.	n.r.	n.r.	n.r.	8.56	n.r.
Félix et al., 2013a [19]	Soybean meal	0.05	6.6	n.r.	n.r.	n.r.	n.r.	n.r.	n.r.	n.r.	10.74	n.r.
Félix et al., 2013a [19]	Micronized soybeans	0.04	6.6	n.r.	n.r.	n.r.	n.r.	n.r.	n.r.	n.r.	13.03	n.r.
Félix et al., 2013a [19]	Toasted soybeans	0.07	3.1	n.r.	n.r.	n.r.	n.r.	n.r.	n.r.	n.r.	10.31	n.r.
Félix et al., 2013a [19]	Raw soybeans	1.74	45.1	n.r.	n.r.	n.r.	n.r.	n.r.	n.r.	n.r.	54.24	n.r.
Félix et al., 2013b [20]	Soybean meal	0.01	n.r.	n.r.	47.5	26.3	88.3	12.1	0.8	175.0	24.01	68.05
Félix et al., 2013b [20]	^1^ Soybean protein concentrate	0.01	n.r.	n.r.	24.4	10.3	24.6	0	0	59.2	11.01	42.35
Félix et al., 2013b [20]	^2^ Soybean protein concentrate	0.01	n.r.	n.r.	4.8	1.9	4.8	0	0	11.4	16.69	56.61
Félix et al., 2013b [20]	^3^ Hydrolyzed soybean protein concentrate	0.03	n.r.	n.r.	4.5	2.0	4.3	0	0	10.8	21.70	66.02
Félix et al., 2013b [20]	Soybean protein isolate	1.52	n.r.	n.r.	0.1	0	0.3	0	0	0.4	43.52	87.41
Purushotham et al., 2007 * [30]	Raw soybeans	2.00	51.0	n.r.	n.r.	n.r.	n.r.	n.r.	n.r.	n.r.	n.r.	n.r.

^1^ Soybean protein concentrate with 600 g crude protein/kg, ^2^ Soybean protein concentrate with 700 g crude protein/kg, ^3^ Hydrolyzed soybean protein concentrate with 700 g crude protein/kg. * Values were reported on an as-is basis.

**Table 3:** Nutritional composition (g/kg on a dry matter basis, unless otherwise stated) of soybean hulls in various pet food studies. (n.r.: not reported).

Authors	Soybean Hull	^1^ DM	Ash	^2^ CP	^3^ AHEE	Crude Fiber	^4^ NDF	^5^ ADF	^6^ TDF	^7^ IF	^8^ SF
Burkhalter et al., 2001 [31]	Soybean hulls (Cargill)	913	53	92	n.r.	n.r.	n.r.	n.r.	773	700	73
Burkhalter et al., 2001 [31]	Soybean hulls (Central Soya)	920	49	123	n.r.	n.r.	n.r.	n.r.	807	722	85
Burkhalter et al., 2001 [31]	Soybean hulls (Jones A)	913	51	130	n.r.	n.r.	n.r.	n.r.	764	637	127
Burkhalter et al., 2001 [31]	Soybean hulls (Jones B)	913	57	149	n.r.	n.r.	n.r.	n.r.	755	684	71
Burkhalter et al., 2001 [31]	Soybean hulls (Quincy)	947	53	155	n.r.	n.r.	n.r.	n.r.	638	599	39
Sabchuk et al., 2017 [32]	Soybean hulls	898	n.r.	130	58.8	384.5	834.8	n.r.	721	655	66
Sabchuk et al., 2014 [33]	Soybean hulls (as-fed basis)	n.r.	n.r.	130	n.r.	n.r.	n.r.	n.r.	720	655	65

^1^ DM = dry matter, ^2^ CP = crude protein, ^3^ AHEE = acid hydrolyzed ether extract, ^4^ NDF = neutral detergent fiber, ^5^ ADF = acid detergent fiber, ^6^ TDF = total dietary fiber, ^7^ IF = insoluble fiber, ^8^ SF = soluble fiber.

**Table 4:** Coefficient of apparent total tract digestibility of nutrients, total dietary fiber degradability, and fecal dry matters of dogs fed diets containing soybean ingredients in various studies. (n.r.: not reported).

Authors	Soybean Ingredient	Inclusion, %	Animal	ATTD, %						Fecal DM, %
				^1^ DM	^2^ OM	^3^ CP	^4^ AHEE	^5^ TDF	^6^ CF	
Félix et al., 2020 [4]	Raw soybeans	0	Adult dog	82.3	85.6	83.2	89.1	n.r.	n.r.	36.4
Félix et al., 2020 [4]	Raw soybeans	6	Adult dog	81.9	85.6	82.1	90.2	n.r.	n.r.	36.3
Félix et al., 2020 [4]	Raw soybeans	12	Adult dog	81.7	85.5	81.8	90.3	n.r.	n.r.	36.5
Félix et al., 2020 [4]	Raw soybeans	18	Adult dog	81.4	85.2	81.3	90.3	n.r.	n.r.	34.8
Félix et al., 2020 [4]	Raw soybeans	24	Adult dog	81.1	85.0	80.8	90.5	n.r.	n.r.	33.6
Félix et al., 2020 [4]	Raw soybeans	30	Adult dog	80.8	84.1	80.6	91.0	n.r.	n.r.	31.3
Carciofi et al., 2009 [9]	Micronized whole soybean	29.3	Adult cat	82.0	85.0	84.0	90.0	n.r.	n.r.	31.1
Carciofi et al., 2009 [9]	Corn gluten meal	17.2	Adult cat	81.0	85.0	84.0	86.0	n.r.	n.r.	30.9
Carciofi et al., 2009 [9]	Micronized whole soybean	33.5	Adult dog	86.0	89.0	87.0	94.0	n.r.	n.r.	30.9
Carciofi et al., 2009 [9]	Soybean meal	29.5	Adult dog	84.0	88.0	86.0	92.0	n.r.	n.r.	31.8
Carciofi et al., 2009 [9]	Poultry by-product meal	22.8	Adult dog	83.0	88.0	85.0	92.0	n.r.	n.r.	45.4
Clapper et al., 2001 [11]	Soybean meal	44.03	Adult dog	81.8	81.7	83.9	92.5	n.r.	n.r.	n.r.
Clapper et al., 2001 [11]	Soybean flour (Soyafluff 200W)	45.16	Adult dog	79.6	85.6	87.3	95.5	n.r.	n.r.	n.r.
Clapper et al., 2001 [11]	SPC 1 (Profine F)	33.17	Adult dog	79.8	84.4	86.5	93.3	n.r.	n.r.	n.r.
Clapper et al., 2001 [11]	SPC 2 (Profine E)	34.06	Adult dog	82.2	84.3	84.7	93.7	n.r.	n.r.	n.r.
Clapper et al., 2001 [11]	SPC 3 (Soyarich I)	33.99	Adult dog	80.9	86.8	89.3	94.5	n.r.	n.r.	n.r.
Clapper et al., 2001 [11]	Poultry meal	32.76	Adult dog	81.9	84.7	76.9	92.9	n.r.	n.r.	n.r.
Yamka et al., 2003 [12]	Soybean meal	15.1	Adult dog	83.1	n.r.	68.1	n.r.	n.r.	n.r.	38.9
Yamka et al., 2003 [12]	Soybean meal	25.5	Adult dog	75.7	n.r.	68.6	n.r.	n.r.	n.r.	32.8
Yamka et al., 2003 [12]	Soybean meal	36.0	Adult dog	64.4	n.r.	64.3	n.r.	n.r.	n.r.	29.7
Yamka et al., 2003 [12]	Soybean meal	46.1	Adult dog	57.4	n.r.	65.5	n.r.	n.r.	n.r.	26.5
Yamka et al., 2005a [13]	Low-oligosaccharide whole soya beans	40.12	Adult dog	85.9	n.r.	81.8.	n.r.	n.r.	n.r.	33.8
Yamka et al., 2005a [13]	Low-oligosaccharide low-phytate whole soya beans	45.21	Adult dog	85.4	n.r.	82.4	n.r.	n.r.	n.r.	28.8
Yamka et al., 2005a [13]	Soya bean meal	31.73	Adult dog	89.1	n.r.	84.8	n.r.	n.r.	n.r.	28.5
Yamka et al., 2005a [13]	Poultry meal, low-ash	22.38	Adult dog	91.3	n.r.	86.4	n.r.	n.r.	n.r.	48.9
Yamka et al., 2005b [14]	Low-oligosaccharide, low-phytate soybean meal	29.22	Adult dog	87.0	n.r.	n.r.	n.r.	n.r.	n.r.	35.5
Yamka et al., 2005b [14]	Conventional soybean meal	30.85	Adult dog	84.8	n.r.	n.r.	n.r.	n.r.	n.r.	36.4
Yamka et al., 2005b [14]	Low-oligosaccharide, low-phytate whole soybean	45.25	Adult dog	82.7	n.r.	n.r.	n.r.	n.r.	n.r.	35.9
Yamka et al., 2005b [14]	Conventional whole soybean	40.1	Adult dog	83.8	n.r.	n.r.	n.r.	n.r.	n.r.	36.3
Yamka et al., 2006 [15]	Low-oligosaccharide, low-phytate soybean meal + β-mannanase (5 g/kg)	29.26	Adult dog	88.0	n.r.	85.1	n.r.	n.r.	n.r.	32.3
Yamka et al., 2006 [15]	Low-oligosaccharide, low-phytate soybean meal	29.26	Adult dog	88.0	n.r.	85.6	n.r.	n.r.	n.r.	29.8
Yamka et al., 2006 [15]	Soybean meal + β-mannanase (5 g/kg)	30.93	Adult dog	86.5	n.r.	85.2	n.r.	n.r.	n.r.	29.3
Yamka et al., 2006 [15]	Soybean meal	30.93	Adult dog	85.5	n.r.	84.5	n.r.	n.r.	n.r.	29.4
Yamka et al., 2006 [15]	Poultry by-product meal + β-mannanase (5 g/kg)	22.43	Adult dog	91.3	n.r.	86.9	n.r.	n.r.	n.r.	40.7
Yamka et al., 2006 [15]	Poultry by-product meal	22.43	Adult dog	91.3	n.r.	86.8	n.r.	n.r.	n.r.	39.6
Pawar and Pattanaik, 2009 [16]	Soybean meal	50	Adult dog	84.2	86.2	94.0	87.7	n.r.	37.1	27.1
Pawar and Pattanaik, 2009 [16]	Soya nugget	50	Adult dog	86.0	87.0	96.1	91.6	n.r.	63.7	23.8
Pattanaik and Kore, 2021 [17]	Soybean meal (twice daily)	30	Adult dog	86.1	99.5	94.9	97.1	n.r.	43.9	27.4
Pattanaik and Kore, 2021 [17]	Soybean meal (once daily)	30	Adult dog	86.1	87.8	95.2	97.3	n.r.	43.0	25.2
Félix et al., 2012 [18]	Regular soybean meal	0	Adult dog	79.3	86.3	81.1	94.7	n.r.	n.r.	41.6
Félix et al., 2012 [18]	Regular soybean meal	15	Adult dog	81.7	86.1	84.5	92.6	n.r.	n.r.	35.5
Félix et al., 2012 [18]	Regular soybean meal	30	Adult dog	80.6	84	84.1	90.7	n.r.	n.r.	29.8
Félix et al., 2012 [18]	High-protein soybean meal	0	Adult dog	79.9	86.1	81.4	94.5	n.r.	n.r.	41.2
Félix et al., 2012 [18]	High-protein soybean meal	15	Adult dog	81.9	86.3	83.6	92.7	n.r.	n.r.	34.8
Félix et al., 2012 [18]	High-protein soybean meal	30	Adult dog	82.6	86.2	84.5	91.9	n.r.	n.r.	29.0
Félix et al., 2013a [19]	Defatted soybean meal	30	Adult dog	75.6	n.r.	85.1	84.3	n.r.	n.r.	31.5
Félix et al., 2013a [19]	Soybean meal	30	Adult dog	75.8	n.r.	85.2	84.3	n.r.	n.r.	31.1
Félix et al., 2013a [19]	Micronized soybeans	30	Adult dog	85.1	n.r.	88.4	96.8	n.r.	n.r.	31.5
Félix et al., 2013a [19]	Toasted soybeans	30	Adult dog	76.7	n.r.	84.7	96.6	n.r.	n.r.	31.5
Félix et al., 2013a [19]	Raw soybeans	30	Adult dog	75.9	n.r.	78.9	96.4	n.r.	n.r.	31.9
Félix et al., 2013a [19]	Defatted soybean meal	30	Puppy	78.3	n.r.	84.8	93.9	n.r.	n.r.	28.2
Félix et al., 2013a [19]	Soybean meal	30	Puppy	77.3	n.r.	85.2	95.8	n.r.	n.r.	28.7
Félix et al., 2013a [19]	Micronized soybeans	30	Puppy	85.0	n.r.	87.4	98.2	n.r.	n.r.	29.4
Félix et al., 2013a [19]	Toasted soybeans	30	Puppy	78.4	n.r.	84.5	98.5	n.r.	n.r.	28.3
Félix et al., 2013a [19]	Raw soybeans	30	Puppy	75.6	n.r.	76.4	99.0	n.r.	n.r.	28.7
Félix et al., 2013b [20]	Soybean meal	30	Adult dog	85.2	84.7	89.8	86.6	n.r.	n.r.	31.5
Félix et al., 2013b [20]	Soybean protein concentrate 600, 600 g crude protein/kg	30	Adult dog	76.5	78.6	83.9	84.5	n.r.	n.r.	29.8
Félix et al., 2013b [20]	Soybean protein concentrate 700, 700 g crude protein/kg	30	Adult dog	77.2	78.4	85.2	85.4	n.r.	n.r.	42.2
Félix et al., 2013b [20]	Hydrolyzed soybean protein concentrate 700, 700 g crude protein/kg	30	Adult dog	86.2	85.5	90.6	87.9	n.r.	n.r.	30.9
Félix et al., 2013b [20]	Soybean protein isolate	30	Adult dog	91.6	92.5	98.8	81.7	n.r.	n.r.	31.4
Tortola et al., 2013 [21] exp1	Soybean meal	30	Adult dog	84.5	86.9	87.0	91.3	59.5	n.r.	30.7
Tortola et al., 2013 [21] exp1	Soybean meal (after extrusion and drying—7500 U protease/kg and 45 U cellulase/kg)	30	Adult dog	83.6	85.8	86.4	91.8	57.2	n.r.	32.1
Tortola et al., 2013 [21] exp1	Soybean meal (after extrusion and drying—15,000 U protease/kg and 90 U cellulase/kg)	30	Adult dog	83.7	86.4	85.8	91.9	60.8	n.r.	28.5
Tortola et al., 2013 [21] exp1	Poultry meal	28.9	Adult dog	85.6	87.6	85.9	91.7	63.0	n.r.	37.0
Tortola et al., 2013 [21] exp2	Soybean meal	30	Adult dog	79.8	83.9	80.5	91.6	49.6	n.r.	30.6
Tortola et al., 2013 [21] exp2	Soybean meal (after extrusion and drying—140 U protease/kg; 8 U cellulase/kg, 800 U pectinase/kg, 60 U phytase/kg, 40 U betaglucanase/kg, 20 U xylanase/kg)	30	Adult dog	80.9	84.5	81.4	93.6	49.9	n.r.	29.2
Tortola et al., 2013 [21] exp2	Soybean meal (after extrusion and drying—700 U protease/kg, 40 U cellulase/kg, 4000 U pectinase/kg, 300 U phytase/kg, 200 U betaglucanase/kg, and 100 U xylanase/kg)	30	Adult dog	80.0	84.1	81.6	93.2	47.3	n.r.	31.6
Tortola et al., 2013 [21] exp2	Poultry meal	28.9	Adult dog	79.1	84.8	79.8	92.8	55.2	n.r.	41.7
Beloshapka et al., 2016 [22]	Bioprocessed soybean protein (HP300)	0	Adult dog	83.0	88.5	82.9	95.3	n.r.	n.r.	41.7
Beloshapka et al., 2016 [22]	Bioprocessed soybean protein (HP300)	4	Adult dog	84.6	89.4	85.8	94.4	n.r.	n.r.	39.4
Beloshapka et al., 2016 [22]	Bioprocessed soybean protein (HP300)	8	Adult dog	84.7	89.2	86.2	95.5	n.r.	n.r.	34.7
Beloshapka et al., 2016 [22]	Bioprocessed soybean protein (HP300)	12	Adult dog	82.2	87.4	84.4	95.4	n.r.	n.r.	34.9
Beloshapka et al., 2016 [22]	Bioprocessed soybean protein (HP300)	24	Adult dog	81.2	86.2	84.5	95.0	n.r.	n.r.	27.0
Beloshapka et al., 2016 [22]	Bioprocessed soybean protein (HP300)	48	Adult dog	77.5	82.6	86.0	93.4	n.r.	n.r.	28.6
Dhaliwal et al., 2016 [24]	soybean meal	34.9	Adult dog	85.5	78.6	82.8	69.1	n.r.	34.8	n.r.
Venturini et al., 2018 [23]	Soybean protein concentrate, coarse particle size	45	Adult dog	81.6	84.6	86.9	91.4	n.r.	n.r.	35.1
Venturini et al., 2018 [23]	Soybean protein concentrate, small particle size	45	Adult dog	82.2	85.6	87.5	92.6	n.r.	n.r.	41.0
Venturini et al., 2018 [23]	Poultry by-product meal	30.9	Adult dog	82.5	86.1	87.8	90.7	n.r.	n.r.	41.8
Venturini et al., 2018 [23]	Corn gluten meal	18.7	Adult dog	83.3	86.6	88.8	92.1	n.r.	n.r.	41.3
Hill et al., 2011 [25]	Texturized soy protein ^7^	0	Dog	n.r.	n.r.	n.r.	n.r.	n.r.	n.r.	31.0
Hill et al., 2011 [25]	Texturized soy protein ^7^	14	Dog	n.r.	n.r.	n.r.	n.r.	n.r.	n.r.	30.0
Hill et al., 2011 [25]	Texturized soy protein ^7^	29	Dog	n.r.	n.r.	n.r.	n.r.	n.r.	n.r.	28.0
Hill et al., 2011 [25]	Texturized soy protein ^7^	57	Dog	n.r.	n.r.	n.r.	n.r.	n.r.	n.r.	25.0
Hill et al., 2011 [25]	Texturized soy protein ^8^	0	Dog	n.r.	n.r.	n.r.	n.r.	n.r.	n.r.	29.0
Hill et al., 2011 [25]	Texturized soy protein ^8^	14	Dog	n.r.	n.r.	n.r.	n.r.	n.r.	n.r.	21.0
Hill et al., 2011 [25]	Texturized soy protein ^8^	29	Dog	n.r.	n.r.	n.r.	n.r.	n.r.	n.r.	19.0
Hill et al., 2011 [25]	Texturized soy protein ^8^	57	Dog	n.r.	n.r.	n.r.	n.r.	n.r.	n.r.	16.0
Menniti et al., 2014 [26]	Soybean meal	0	Adult dog	81.1	85.0	81.3	91.2	n.r.	n.r.	32.7
Menniti et al., 2014 [26]	Soybean meal	6	Adult dog	80.2	83.9	80.9	91.0	n.r.	n.r.	32.4
Menniti et al., 2014 [26]	Soybean meal	11.5	Adult dog	80.9	84.5	82.1	91.8	n.r.	n.r.	30.8
Menniti et al., 2014 [26]	Soybean meal	17	Adult dog	81.4	85.0	83.1	92.0	n.r.	n.r.	30.2
Marx et al., 2015 [27]	Soybean oil	0	Adult dog	68.7	77.7	80.8	86.3	n.r.	n.r.	34.7
Marx et al., 2015 [27]	Soybean oil	6.5	Adult dog	70.4	78.9	80.8	78.9	n.r.	n.r.	35.1
Marx et al., 2015 [27]	Soybean oil	13	Adult dog	73.4	81.1	81.4	79.8	n.r.	n.r.	35.8
Kaur et al., 2021 * [34]	Soybean nugget	0	N/A	92.2	91.3	92.8	94.9	n.r.	n.r.	n.r.
Kaur et al., 2021 * [34]	Soybean nugget	5	N/A	88.2	87.9	88.1	88.9	n.r.	n.r.	n.r.
Kaur et al., 2021 * [34]	Soybean nugget	10	N/A	89.3	89.9	89.7	91.8	n.r.	n.r.	n.r.
Kaur et al., 2021 * [34]	Soybean nugget	15	N/A	88.4	88.3	89.0	90.2	n.r.	n.r.	n.r.
Hill et al., 2001 [36]	Texturized soy protein	0	Adult dog	87.0	n.r.	86.3	98.9	n.r.	n.r.	39.0
Hill et al., 2001 [36]	Texturized soy protein	14	Adult dog	86.9	n.r.	84.0	98.9	n.r.	n.r.	36.0
Hill et al., 2001 [36]	Texturized soy protein	29	Adult dog	85.9	n.r.	83.1	98.9	n.r.	n.r.	34.0
Hill et al., 2001 [36]	Texturized soy protein	57	Adult dog	83.9	n.r.	80.1	98.6	n.r.	n.r.	28.0

^1^ DM = dry matter, ^2^ OM = organic matter, ^3^ CP = crude protein, ^4^ AHEE = acid hydrolyzed ether extract, ^5^ TDF = total dietary fiber, ^6^ CF = crude fiber, ^7^ Dogs were morning-fed, ^8^ Dogs were afternoon-fed. * in vitro digestibility values for extruded dog feed.

**Table 5:** Coefficient of ileal digestibility (small intestine digestibility or pre-cecal digestibility) in dogs fed soybean-included diets. (n.r.: not reported).

Authors	Soybean Ingredient	Inclusion, %	Animal	Apparent Ileal Digestibility, %		
				^1^ DM	^2^ CP	^3^ AHEE
Yamka et al., 2003 [12]	Soybean meal	15.1	Adult dog	80.7	65.4	n.r.
Yamka et al., 2003 [12]	Soybean meal	25.5	Adult dog	71.0	66.2	n.r.
Yamka et al., 2003 [12]	Soybean meal	36.0	Adult dog	53.4	59.8	n.r.
Yamka et al., 2003 [12]	Soybean meal	46.1	Adult dog	33.8	51.1	n.r.
Yamka et al., 2005a [13]	Low-oligosaccharide whole soya beans	40.12	Adult dog	78.0	71.7	n.r.
Yamka et al., 2005a [13]	Low-oligosaccharide low-phytate whole soya beans	45.21	Adult dog	76.4	75.1	n.r.
Yamka et al., 2005a [13]	Soya bean meal	31.73	Adult dog	80.8	78.2	n.r.
Yamka et al., 2005a [13]	Low-ash poultry meal	22.38	Adult dog	86.2	72.9	n.r.
Yamka et al., 2005b [14]	Low-oligosaccharide, low-phytate soybean meal	29.22	Adult dog	80.9	79.2	n.r.
Yamka et al., 2005b [14]	Conventional soybean meal	30.85	Adult dog	77.5	82.0	n.r.
Yamka et al., 2005b [14]	Low-oligosaccharide, low-phytate whole soybean	45.25	Adult dog	74.0	68.8	n.r.
Yamka et al., 2005b [14]	Conventional whole soybean	40.1	Adult dog	76.1	69.8	n.r.
Hill et al., 2000 [35]	Texturized soybean protein	0	Adult dog	80.6	77.0	99.4
Hill et al., 2000 [35]	Texturized soybean protein	14	Adult dog	77.1	73.4	99.5
Hill et al., 2000 [35]	Texturized soybean protein	29	Adult dog	75.2	71.8	98.8
Hill et al., 2000 [35]	Texturized soybean protein	57	Adult dog	71.7	70.8	99.4
Hill et al., 2001 [36]	Texturized soybean protein	0	Adult dog	80.6	77.0	99.4
Hill et al., 2001 [36]	Texturized soybean protein	14	Adult dog	77.1	73.4	99.5
Hill et al., 2001 [36]	Texturized soybean protein	29	Adult dog	75.2	71.8	98.8
Hill et al., 2001 [36]	Texturized soybean protein	57	Adult dog	71.7	70.8	99.4

^1^ DM = dry matter, ^2^ CP = crude protein, ^3^ AHEE = acid hydrolyzed ether extract.

**Table 6:** Coefficient of apparent total tract digestibility, total dietary fiber degradability, and fecal dry matter (%) in companion animals fed diets containing soybean hulls. (n.r.: not reported).

Authors	Soybean Hull	Inclusion, %	Animal	ATTD, %					Fecal DM, %
				^1^ DM	^2^ OM	^3^ CP	^4^ AHEE	^5^ TDF	
Detweiler et al., 2019b [10]	Soybean hull	0	Adult cat	85.5	88.8	84.9	89.9	8.5	35.0
Detweiler et al., 2019b [10]	Soybean hull	14	Adult cat	75.4	78.5	81.7	88.6	18.0	38.9
Burkhalter et al., 2001 [31]	Soybean hulls (Central Soya)	7.5	Adult dog	72.7	79.0	74.1	92.6	−8.5	36.0
Burkhalter et al., 2001 [31]	Soybean hulls (Cargill)	7.5	Adult dog	70.9	78.0	72.7	92.0	−9.6	37.0
Burkhalter et al., 2001 [31]	Soybean hulls (Jones-A)	7.5	Adult dog	74.6	81.7	78.4	94.0	11.6	36.0
Burkhalter et al., 2001 [31]	Soybean hulls (Quincy)	7.5	Adult dog	69.2	77.0	70.9	91.5	−7.3	32.0
Burkhalter et al., 2001 [31]	Soybean hulls (Jones-B)	7.5	Adult dog	71.3	78.4	73.9	92.2	−10.4	36.0
Sabchuk et al., 2017 [32]	Soybean hull	0	Adult dog	84.0	n.r.	88.9	91.1	n.r.	37.1
Sabchuk et al., 2017 [32]	Soybean hull	4	Adult dog	80.1	n.r.	85.2	85.7	n.r.	31.1
Sabchuk et al., 2017 [32]	Soybean hull	8	Adult dog	78.8	n.r.	86.4	89.5	n.r.	35.9
Sabchuk et al., 2017 [32]	Soybean hull	12	Adult dog	73.8	n.r.	84.2	86.8	n.r.	34.4
Sabchuk et al., 2017 [32]	Soybean hull	16	Adult dog	71.9	n.r.	83.4	85.9	n.r.	35.4
Detweiler et al., 2019a [37]	Soybean hull	0	Adult dog	85.4	90.1	85.8	90.9	37.8	44.7
Detweiler et al., 2019a [37]	Soybean hull	15	Adult dog	79.6	79.9	83.3	91.9	22.7	39.4

^1^ DM = dry matter, ^2^ OM = organic matter, ^3^ CP = crude protein, ^4^ AHEE = acid hydrolyzed ether extract, ^5^ TDF = total dietary fiber.

**Table 7:** Fresh fecal pH, ammonia, short-chain fatty acids (SCFA), branched-chain plus minor fatty acids (BCMFA), lactate, phenol, and indole concentrations in fecal samples from dogs fed soybean diets presented in μmol/g DM (different units specified with footnotes). (n.r.: not reported).

Authors	Soybean Ingredient	Inclusion, %	Animal	Fecal pH	SCFA				BCMFA				Lactate	Ammonia	Phenol	Indole
					Acetate	Propionate	Butyrate	^1^ Total SCFA	Valerate	Isobutyrate	Isovalerate	^2^ Total BCMFA				
Félix et al., 2020 [4]	Raw soybeans	6	dog	6.4	n.r.	n.r.	n.r.	n.r.	n.r.	n.r.	n.r.	n.r.	n.r.	n.r.	n.r.	n.r.
Félix et al., 2020 [4]	Raw soybeans	12	dog	6.5	n.r.	n.r.	n.r.	n.r.	n.r.	n.r.	n.r.	n.r.	n.r.	n.r.	n.r.	n.r.
Félix et al., 2020 [4]	Raw soybeans	18	dog	6.5	n.r.	n.r.	n.r.	n.r.	n.r.	n.r.	n.r.	n.r.	n.r.	n.r.	n.r.	n.r.
Félix et al., 2020 [4]	Raw soybeans	24	dog	6.6	n.r.	n.r.	n.r.	n.r.	n.r.	n.r.	n.r.	n.r.	n.r.	n.r.	n.r.	n.r.
Félix et al., 2020 [4]	Raw soybeans	30	dog	6.6	n.r.	n.r.	n.r.	n.r.	n.r.	n.r.	n.r.	n.r.	n.r.	n.r.	n.r.	n.r.
Pawar and Pattanaik, 2009 [16]	Soybean meal	50	dog	4.9	260.6	170.4	39.8	470.7	n.r.	n.r.	n.r.	n.r.	14.6	10.5	n.r.	n.r.
Pawar and Pattanaik, 2009 [16]	Soya nugget	50	dog	5.1	121.8	65.3	27.4	214.6	n.r.	n.r.	n.r.	n.r.	7.9	6.1	n.r.	n.r.
Pattanaik and Kore, 2021 [17]	Soybean meal (twice daily)	30	dog	5.2	n.r.	n.r.	n.r.	n.r.	n.r.	n.r.	n.r.	n.r.	n.r.	n.r.	n.r.	n.r.
Pattanaik and Kore, 2021 [17]	Soybean meal (once daily)	30	dog	5.4	n.r.	n.r.	n.r.	n.r.	n.r.	n.r.	n.r.	n.r.	4.5	n.r.	n.r.	n.r.
Félix et al., 2012 [18]	Soybean meal	15	dog	6.6	n.r.	n.r.	n.r.	n.r.	n.r.	n.r.	n.r.	n.r.	n.r.	n.r.	n.r.	n.r.
Félix et al., 2012 [18]	Soybean meal	30	dog	6.3	n.r.	n.r.	n.r.	n.r.	n.r.	n.r.	n.r.	n.r.	n.r.	n.r.	n.r.	n.r.
Félix et al., 2012 [18]	High-protein soybean meal	15	dog	6.7	n.r.	n.r.	n.r.	n.r.	n.r.	n.r.	n.r.	n.r.	n.r.	n.r.	n.r.	n.r.
Félix et al., 2012 [18]	High-protein soybean meal	30	dog	6.5	n.r.	n.r.	n.r.	n.r.	n.r.	n.r.	n.r.	n.r.	n.r.	n.r.	n.r.	n.r.
Félix et al., 2013a [19]	Defatted soybean meal	30	dog	5.9	n.r.	n.r.	n.r.	n.r.	n.r.	n.r.	n.r.	n.r.	n.r.	230.2	n.r.	n.r.
Félix et al., 2013a [19]	Soybean meal	30	dog	5.8	n.r.	n.r.	n.r.	n.r.	n.r.	n.r.	n.r.	n.r.	n.r.	220.2	n.r.	n.r.
Félix et al., 2013a [19]	Micronized soybeans	30	dog	5.9	n.r.	n.r.	n.r.	n.r.	n.r.	n.r.	n.r.	n.r.	n.r.	219.6	n.r.	n.r.
Félix et al., 2013a [19]	Toasted soybeans	30	dog	6.9	n.r.	n.r.	n.r.	n.r.	n.r.	n.r.	n.r.	n.r.	n.r.	227.8	n.r.	n.r.
Félix et al., 2013a [19]	Raw soybeans	30	dog	6.9	n.r.	n.r.	n.r.	n.r.	n.r.	n.r.	n.r.	n.r.	n.r.	259.0	n.r.	n.r.
Félix et al., 2013a [19]	Defatted soybean meal	30	puppy	5.6	n.r.	n.r.	n.r.	n.r.	n.r.	n.r.	n.r.	n.r.	n.r.	307.1	n.r.	n.r.
Félix et al., 2013a [19]	Soybean meal	30	puppy	5.6	n.r.	n.r.	n.r.	n.r.	n.r.	n.r.	n.r.	n.r.	n.r.	299.5	n.r.	n.r.
Félix et al., 2013a [19]	Micronized soybeans	30	puppy	5.6	n.r.	n.r.	n.r.	n.r.	n.r.	n.r.	n.r.	n.r.	n.r.	303.0	n.r.	n.r.
Félix et al., 2013a [19]	Toasted soybeans	30	puppy	6.6	n.r.	n.r.	n.r.	n.r.	n.r.	n.r.	n.r.	n.r.	n.r.	296.5	n.r.	n.r.
Félix et al., 2013a [19]	Raw soybeans	30	puppy	6.6	n.r.	n.r.	n.r.	n.r.	n.r.	n.r.	n.r.	n.r.	n.r.	330.0	n.r.	n.r.
Félix et al., 2013b [20]	Soybean meal	30	dog	6.5	n.r.	n.r.	n.r.	n.r.	n.r.	n.r.	n.r.	n.r.	n.r.	146.2 *	n.r.	n.r.
Félix et al., 2013b [20]	Soybean protein concentrate 600, 600 g crude protein/kg	30	dog	6.6	n.r.	n.r.	n.r.	n.r.	n.r.	n.r.	n.r.	n.r.	n.r.	158.0 *	n.r.	n.r.
Félix et al., 2013b [20]	Soybean protein concentrate 700, 700 g crude protein/kg	30	dog	6.4	n.r.	n.r.	n.r.	n.r.	n.r.	n.r.	n.r.	n.r.	n.r.	160.3 *	n.r.	n.r.
Félix et al., 2013b [20]	Hydrolyzed soybean protein concentrate 700, 700 g crude protein/kg	30	dog	6.5	n.r.	n.r.	n.r.	n.r.	n.r.	n.r.	n.r.	n.r.	n.r.	157.4 *	n.r.	n.r.
Félix et al., 2013b [20]	Soybean protein isolate	30	dog	7.0	n.r.	n.r.	n.r.	n.r.	n.r.	n.r.	n.r.	n.r.	n.r.	153.3 *	n.r.	n.r.
Tortola et al., 2013 [21] exp1	Soybean meal	30	dog	6.2	272.0	244.0	22.0	539.0	n.r.	n.r.	n.r.	n.r.	16.7	76.3	n.r.	n.r.
Tortola et al., 2013 [21] exp1	Soybean meal ^3^	30	dog	6.3	246.0	208.0	33.0	488.0	n.r.	n.r.	n.r.	n.r.	17.0	70.5	n.r.	n.r.
Tortola et al., 2013 [21] exp1	Soybean meal ^4^	30	dog	6.1	246.0	196.0	27.0	470.0	n.r.	n.r.	n.r.	n.r.	18.8	88.1	n.r.	n.r.
Tortola et al., 2013 [21] exp2	Soybean meal	30	dog	5.9	324.0	245.0	56.8	324.0	n.r.	n.r.	n.r.	n.r.	20.1	70.5	n.r.	n.r.
Tortola et al., 2013 [21] exp2	Soybean meal ^5^	30	dog	5.9	356.0	313.0	41.9	356.0	n.r.	n.r.	n.r.	n.r.	29.2	70.5	n.r.	n.r.
Tortola et al., 2013 [21] exp2	Soybean meal ^6^	30	dog	6.0	370.0	306.0	43.1	370.0	n.r.	n.r.	n.r.	n.r.	16.8	76.3	n.r.	n.r.
Beloshapka et al., 2016 [22]	Biopressed soybean protein (HP300)	4	dog	6.6	162.0	93.4	39.2	294.6	0.7	7.8	12.4	20.9	n.r.	117.7	2.2	2.2
Beloshapka et al., 2016 [22]	Biopressed soybean protein (HP300)	8	dog	6.4	180.8	96.0	32.3	309.1	0.6	5.8	9.0	15.3	n.r.	116.6	1.2	2.2
Beloshapka et al., 2016 [22]	Biopressed soybean protein (HP300)	12	dog	6.2	224.2	109.8	46.3	380.3	0.7	6.0	9.0	15.7	n.r.	114.7	1.1	1.8
Beloshapka et al., 2016 [22]	Biopressed soybean protein (HP300)	24	dog	6.3	367.7	177.8	48.3	593.7	1.3	5.9	8.5	15.7	n.r.	134.2	0.7	0.7
Beloshapka et al., 2016 [22]	Biopressed soybean protein (HP300)	48	dog	6.4	318.0	188.8	40.0	546.7	1.2	3.5	5.1	9.9	n.r.	70.5	0.3	0.8
Venturini et al., 2018 [23]	Soybean protein concentrate, coarse particle size	45	dog	6.2	n.r.	n.r.	n.r.	n.r.	n.r.	n.r.	n.r.	n.r.	4.1	94	n.r.	n.r.
Venturini et al., 2018 [23]	Soybean protein concentrate, small particle size	45	dog	6.4	n.r.	n.r.	n.r.	n.r.	n.r.	n.r.	n.r.	n.r.	n.r.	102	n.r.	n.r.

^1^ Total SCFA = Acetate + propionate = butyrate, ^2^ Total BCMFA = Valerate + isobutyrate + isovalerate, ^3^ Soybean meal (after extrusion and drying—7500 U protease/kg and 45 U cellulase/kg), ^4^ Soybean meal (after extrusion and drying—15,000 U protease/kg and 90 U cellulase/kg), ^5^ Soybean meal (after extrusion and drying—140 U protease/kg; 8 U cellulase/kg, 800 U pectinase/kg, 60 U phytase/kg, 40 U betaglucanase/kg, 20 U xylanase/kg), ^6^ Soybean meal (after extrusion and drying—700 U protease/kg, 40 U cellulase/kg, 4000 U pectinase/kg, 300 U phytase/kg, 200 U betaglucanase/kg and 100 U xylanase/kg). * Ammonia concentration μmol/g as-is fecal sample.

**Table 8:** Fresh fecal pH, ammonia, short-chain fatty acids (SCFA), branched-chain plus minor fatty acids (BCMFA), lactate, phenol, indole, and skatole concentrations in fecal samples from companion animals fed soybean hulls, presented in μmol/g DM unless otherwise specified with footnotes. (n.r.: not reported).

Authors	Soybean Hull	Inclusion, %	Animal	Fecal pH	SCFA				BCMFA				Ammonia	Phenol	Indole
					Acetate	Propionate	Butyrate	^1^ Total SCFA	Valerate	Isobutyrate	Isovalerate	^2^ Total BCMFA			
Detweiler et al., 2019b [10]	soybean hull	14	cat	5.7	274.3	76.2	72.1	422.7	5.8	7.6	13.4	26.8	130.5	0.5	1.4
Sabchuk et al., 2017 [32]	Soybean hull	4	dog	5.9	n.r.	n.r.	n.r.	n.r.	n.r.	n.r.	n.r.	n.r.	34.6 *	n.r.	n.r.
Sabchuk et al., 2017 [32]	Soybean hull	8	dog	6.8	n.r.	n.r.	n.r.	n.r.	n.r.	n.r.	n.r.	n.r.	59.9 *	n.r.	n.r.
Sabchuk et al., 2017 [32]	Soybean hull	12	dog	6.6	n.r.	n.r.	n.r.	n.r.	n.r.	n.r.	n.r.	n.r.	46.4 *	n.r.	n.r.
Sabchuk et al., 2017 [32]	Soybean hull	16	dog	6.6	n.r.	n.r.	n.r.	n.r.	n.r.	n.r.	n.r.	n.r.	54.6 *	n.r.	n.r.
Detweiler et al., 2019a [37]	Soybean hull	15	dog	5.9	321.0	121.0	37.7	479.7	0.9	6.3	9.8	17.0	147.8	0.3	1.4
Myint et al., 2017 [41]	Soybean husk	5.6	dog	5.9	91.2	54.5	10.6	158.3	n.r.	n.r.	n.r.	n.r.	0.1	n.r.	4.1

^1^ Total SCFA = Acetate + propionate = butyrate, ^2^ Total BCMFA = Valerate + isobutyrate + isovalerate. * Ammonia concentration μmol/g as-is fecal sample.

## Data Availability

Not applicable.
